
JOURNAL INFORMATION
==============================
NLM Title Abbreviation: ScientificWorldJournal
Journal ID: ScientificWorldJournal
NLM Journal ID: 101131163
Journal ID: TSWJ

The Scientific World Journal

ISSN: 2356-6140
EISSN: 1537-744X
Publisher: Wiley

ARTICLE INFORMATION
==============================
PMCID: PMC6084049
PMID: 30147626
DOI: 10.1100/tsw.2001.23.248
Article ID: 574085
Article version: 1
Subjects: Short Report

Muc4\sialomucin Complex, A Specific Intramembrane Modulator of ERBB2/HER2/NEU, Potentiates Primary Tumor Growth and Suppresses Apoptosis in A Xenotransplanted Melanoma

Carraway Coralie Carothers
Komatsu Masanobu
Jepson Scott
Arango Maria
Carraway Kermit
Publication date: 2001
Electronic publication date: 2001 Oct 11
Volume: 1
First page: 149
Last page: 149
Copyright: Copyright © 2001 Coralie Carothers Carraway et al.
Copyright year: 2001
License: This is an open access article distributed under the Creative Commons Attribution License, which permits unrestricted use, distribution, and reproduction in any medium, provided the original work is properly cited.
License URL: https://creativecommons.org/licenses/by/3.0/

==============================
Miami *Nature Biotechnology* Short Reports
*TheScientificWorld* (2001) 1, 149SR
ISSN 1532-2246

### MUC4/SIALOMUCIN COMPLEX, A SPECIFIC INTRAMEMBRANE MODULATOR OF ERBB2/HER2/NEU, POTENTIATES PRIMARY TUMOR GROWTH AND SUPPRESSES APOPTOSIS IN A XENOTRANSPLANTED MELANOMA

Coralie Carothers Carraway[1], Masanobu Komatsu[2], Scott Jepson[2], Maria Arango[2], and Kermit Carraway[2]

Depts. of Biochemistry & Molecular Biology[1] and Cell Biology & Anatomy[2], University of Miami School of Medicine, Miami, FL 33101

**INTRODUCTION.** Sialomucin complex (SMC/MUC4) is a large mucin-containing complex implicated in tumor progression. Overexpression of the membrane mucin MUC4/Sialomucin complex (SMC) has been observed during malignant progression of mammary tumors in both humans and rats, suggesting that deregulation of MUC4/SMC expression facilitates development of these malignancies. The complex, expressed preferentially in epithelial tissues and their tumors, was originally isolated from and characterized in the aggressive 13762 ascites rat mammary adenocarcinoma.[1] SMC/Muc4 is a large, heterodimeric glycoprotein complex composed of two subunits, a heavily glycosylated mucin extracellular domain ASGP-1, stably associated with the N-glycosylated transmembrane subunit ASGP-2. The ASGP-2 sequence contains two EGF-like domains within the extracellular region, suggesting a potential ligand role; the characterization of an ASGP-2/ErbB2-containing signal transduction particle in the constitutively activated ascites cells[2] provided support for this ligand hypothesis. Co-infection of insect cells with ASGP-2 and members of the EGFR family demonstrated that ASGP-2 binds selectively via EGF-1 to ErbB2, up-regulating the receptor's kinase activity and receptor autophosphorylation; and modulating the effects of the soluble ligand NDF/HRG on the ErbB2/ErbB3 heterodimer.[3] Previous studies have shown that overexpression of SMC in A375 melanoma cells causes increased tumor metastases when the cells were injected into nude mice.[4] The present studies attempt to elucidate the role(s) of SMC in the potentiation of tumor cell survival and to determine the effect of overexpression of SMC on apoptosis in serum starved A375 melanoma cells.

**METHODS.**

*In vivo tumor growth analyses.* A375 melanoma cells transfected with an inducible expression of SMC (Rep8) were described previously.[4,5] The *in vivo* SMC expression was analyzed by FACScan analyses and immunohistochemical staining of paraffin sections using anti-SMC mAb 4F12.

*In vitro effects of SMC expression.* A375 melanoma cells stably transfected with a tetracycline regulatable SMC expression vector were grown in medium containing 10% FCS. SMC overexpression was induced by removal of tetracycline (Tet-Off system) from the culture.[4] *In vitro* expression levels of SMC in untransfected and transfected cells were determined by FACScan analyses. The proliferative potential of SMC transfectants was examined by measuring cell cycle progression in low serum as well as colony formation efficiency in soft agarose. To induce apoptosis the growth medium was replaced with serum free medium supplemented with 0.1% BSA and the cells grown for varying lengths of time. The effect of SMC expression on apoptosis was determined in several ways: 1) by TUNEL assays on paraffin-embedded tumor sections using the DeadEnd™ Apoptosis Detection System (Promega, Madison, WI); 2) flow cytometry using annexin V and propidium iodide; and 3) PARP cleavage by Western blot. Full length PARP was detected as a 115-kDa band. The apoptosis-related cleavage fragment of PARP was approx. 85-90 kDa.

**RESULTS AND DISCUSSION.** The present studies investigated the effect of Muc4/SMC up-regulation on primary tumor growth using a tetracycline-inducible SMC expression system in a xenotransplanted tumor model. SMC up-regulation provoked rapid growth of transfected A375 melanoma in nude mice (Fig. 1). *In vitro* studies on SMC-transfected cells, however, suggested that the effect of SMC on *in vivo* tumor growth was not a consequence of enhanced cell proliferation, since up-regulation of SMC did not significantly increase proliferation of A375 cell. Instead, a strong suppression of apoptosis was observed *in situ* in SMC-overexpressing tumors. Immunoblot analyses with anti-PARP mAb demonstrated that the cleavage of the caspase PARP is reduced in SMC-overexpressing cells (Fig. 2), suggesting the down-regulation of caspase3 and/or caspase9 in these cells. These observations are in agreement with the findings described above and suggest that regulation of these caspase activities is at least one of the targets for SMC-promoted cell survival signaling.

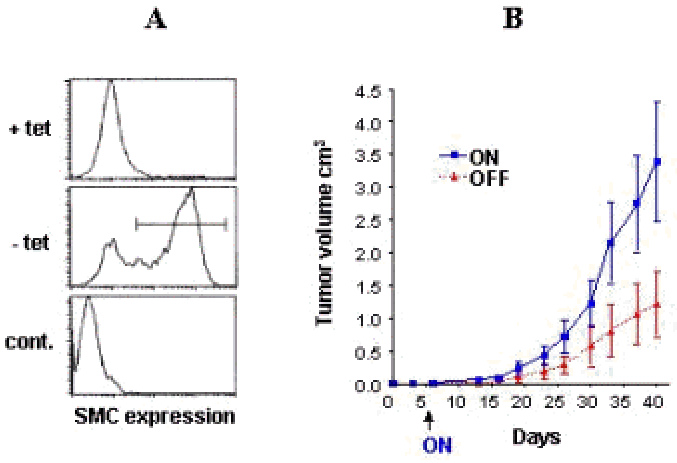

**Figure 1**

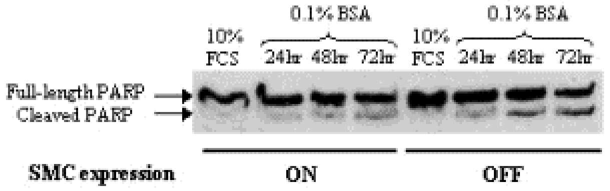

**Figure 2**

**CONCLUSION.** These data suggest that Muc4/SMC expression promotes tumor growth *in vivo* at least in part via suppression of tumor cell apoptosis. Importantly, reduction of apoptosis was also observed *in vitro*, indicating that the anti-apoptotic effect of SMC is independent of tumor-host interactions. These findings strongly suggest that SMC up-regulation alters intracellular signaling to favor cell survival. The combination of *in vivo* and *in vitro* studies on the effects of SMC expression on tumor growth properties strongly suggest that SMC up-regulation alters intracellular signaling to favor cell survival, providing for the first time ***evidence for the regulation of programmed cell death by a gene of the MUC family***.

**REFERENCES.**

1. Sherblom, A.P. and Carraway, K.L. (1980) J. Biol. Chem. 255, 12051-12059
2. Carraway, C.A.C., Carvajal, M.E., and Carraway, K.L. (1999) J. Biol. Chem. 274, 25659-25667
3. Carraway, K.L., III, Rossi, E.A., Komatsu, M., Price-Schiavi, S.A., Huang, D., Carvajal, M.E., Guy, P.M., Fregien, N., Carraway, C.A.C., and Carraway, K.L. (1999) J. Biol. Chem. 274, 5263-5266
4. Komatsu, M., Tatum, L., Altman, N.H., Carraway, C.A.C., and Carraway, K.L. (2000) Int. J. Cancer 87, 480-486
5. Komatsu, M., Carraway, C.A.C., Fregien, N.L., and Carraway, K.L. (1997) J. Biol. Chem. 272, 33245-33254
